# Supplementary material for: Up State of the SARS-COV-2 Spike Homotrimer Favors an Increased Virulence for New Variants
Source: Front Med Technol. 2021 Jun 30;3:694347. doi: 10.3389/fmedt.2021.694347 (PMC8757851; doi:10.3389/fmedt.2021.694347)
Supplement: Supplementary file 1 [file Data_Sheet_1.pdf]

Supplementary Material

1 Supplementary Figures and Tables

Additional details are given as supplementary material for:

1.1 Supplementary Figures

1.1.1 Sequences alignment

Sequences alignment of SARS-CoV-1 and SARS-CoV-2 RBD (S1 subunit) proteins

```
SARS-CoV-1         1 ----CPFGEVFNATKFPSVYAWERKKISNCVADYSVLYNSTFFSTFKCYG     46
                         ||||||||||:|.|||||.||:||||||||||||||..||||||||
SARS-CoV-2         1 ITNLCPFGEVFNATRFASVYAWNRKRISNCVADYSVLYNSASFSTFKCYG     50

SARS-CoV-1        47 VSATKLNDLCFSNVYADSFVVKGDDVRQIAPGQTGVIADYNYKLPDDFMG     96
                     ||.||||||||:||||||||::||:||||||||||.||||||||||||.|
SARS-CoV-2        51 VSPTKLNDLCFTNVYADSFVIRGDEVRQIAPGQTGKIADYNYKLPDDFTG    100

SARS-CoV-1        97 CVLAWNTRNIDATSTGNYNYKYRYLRHGKLRPFERDISNVPFSPDGKPCT    146
                     ||:|||:.|:|:...|||||.||..|...|:|||||||...:.....||.
SARS-CoV-2       101 CVIAWNSNNLDSKVGGNYNYLYRLFRKSNLKPFERDISTEIYQAGSTPCN    150

SARS-CoV-1       147 -PPALNCYWPLNDYGFYTTTGIGYQPYRVVVLSFE-------    180
                      ....|||:||..|||..|.|:|||||||||||||
SARS-CoV-2       151 GVEGFNCYFPLQSYGFQPTNGVGYQPYRVVVLSFELLHAPAT    192
```

Supplementary Figure 1. Sequence alignment of SARS-CoV-1 and SARS-CoV-2 wt RBD (S1 subunit) proteins. The numbers next to the chains are used to guide the identification of the amino acid sequence numbers of the RBD. All sequence numbers were automatically shifted by the EMBOSS Needle server. Original numbers can be recovered by the addition of 331 for the SARS-CoV-2 (for instance, N170 in this alignment corresponds to N501 in the PDB files for this strain). The signs between the aligned sequences have the typical meaning: a) "|" indicates equal amino acids in both sequences; b) ";" indicates similarities with a high score and c) "." indicates the amino acids with a low positive score. Gaps are represented by "-". See text for more information.

Sequences alignment of SARS-CoV-2 and SARS-CoV-2’ RBD (S1 subunit) proteins

```
SARS-CoV-2 wt            1 ITNLCPFGEVFNATRFASVYAWNRKRISNCVADYSVLYNSASFSTFKCYG     50
                           ||||||||||||||||||||||||||||||||||||||||||||||||||
SARS-CoV-2 (B.1.351)     1 ITNLCPFGEVFNATRFASVYAWNRKRISNCVADYSVLYNSASFSTFKCYG     50

SARS-CoV-2 wt           51 VSPTKLNDLCFTNVYADSFVIRGDEVRQIAPGQTGKIADYNYKLPDDFTG    100
                           |||||||||||||||||||||||||||||||||||.||||||||||||||
SARS-CoV-2 (B.1.351)    51 VSPTKLNDLCFTNVYADSFVIRGDEVRQIAPGQTGNIADYNYKLPDDFTG    100

SARS-CoV-2 wt          101 CVIAWNSNNLDSKVGGNYNYLYRLFRKSNLKPFERDISTEIYQAGSTPCN    150
                           ||||||||||||||||||||||||||||||||||||||||||||||||||
SARS-CoV-2 (B.1.351)   101 CVIAWNSNNLDSKVGGNYNYLYRLFRKSNLKPFERDISTEIYQAGSTPCN    150

SARS-CoV-2 wt          151 GVEGFNCYFPLQSYGFQPTNGVGYQPYRVVVLSFELLHAPAT    192
                           ||:||||||||||||||||.||||||||||||||||||||||
SARS-CoV-2 (B.1.351)   151 GVKGFNCYFPLQSYGFQPTYGVGYQPYRVVVLSFELLHAPAT    192
```

Supplementary Figure 2. Sequence alignment of SARS-CoV-2 wt and SARS-CoV-2 (B.1.351) RBD (S1 subunit) proteins. All the other details are as in Fig. S1.

Sequences alignment of SARS-CoV-1 and SARS-CoV-2’ RBD (S1 subunit) proteins

```
SARS-CoV-1               1 ----CPFGEVFNATKFPSVYAWERKKISNCVADYSVLYNSTFFSTFKCYG     46
                               ||||||||||:|.|||||.||:||||||||||||||..||||||||
SARS-CoV-2 (B.1.351)     1 ITNLCPFGEVFNATRFASVYAWNRKRISNCVADYSVLYNSASFSTFKCYG     50

SARS-CoV-1              47 VSATKLNDLCFSNVYADSFVVKGDDVRQIAPGQTGVIADYNYKLPDDFMG     96
                           ||.||||||||:||||||||::||:||||||||||.||||||||||||.|
SARS-CoV-2 (B.1.351)    51 VSPTKLNDLCFTNVYADSFVIRGDEVRQIAPGQTGNIADYNYKLPDDFTG    100

SARS-CoV-1              97 CVLAWNTRNIDATSTGNYNYKYRYLRHGKLRPFERDISNVPFSPDGKPCT    146
                           ||:|||:.|:|:...|||||.||..|...|:|||||||...:.....||.
SARS-CoV-2 (B.1.351)   101 CVIAWNSNNLDSKVGGNYNYLYRLFRKSNLKPFERDISTEIYQAGSTPCN    150

SARS-CoV-1             147 -PPALNCYWPLNDYGFYTTTGIGYQPYRVVVLSFE-------    180
                            ....|||:||..|||..|.|:|||||||||||||
SARS-CoV-2 (B.1.351)   151 GVKGFNCYFPLQSYGFQPTYGVGYQPYRVVVLSFELLHAPAT    192
```

Supplementary Figure 3. Sequence alignment of SARS-CoV-1 and SARS-CoV-2 (B.1.351) RBD (S1 subunit) proteins. All the other details are as in Fig. S1.

1.1.2 RBD-ACE2 Complexation

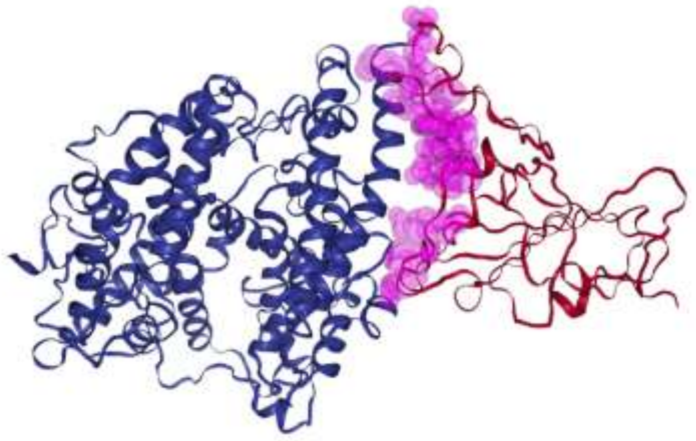

Supplementary Figure 4. Molecular structures of the SARS-CoV-2 S RBD complexed with ACE2. For better visualization of the epitope-paratope interface, the interface residues on the RBD were represented as overpost pink spheres. Interface residues are defined as residues whose atoms are within a distance of 5 Angstroms from other atoms of the neighboring chains. Red represents RBD on SARS-CoV-2 and the blue represents the receptor ACE2 (PDB id 6LZG). The image was generated by CoV3D (90).

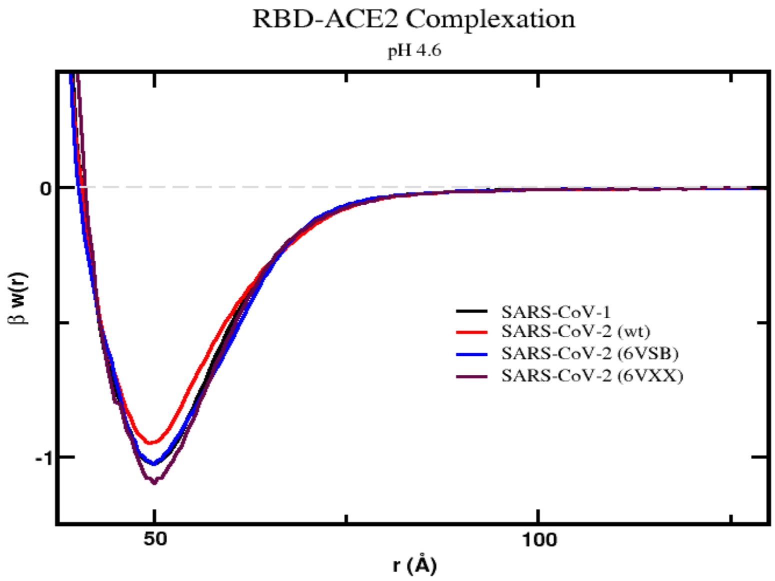

Supplementary Figure 5. Free energy profiles for the interaction of RBD proteins obtained from different structural data with the cellular receptor ACE2. The simulated free energy of interactions [**β***w*(*r*)] between the centers of the RBD proteins from SARS-CoV-1 and different coordinates for the SARS-CoV-2 wildtype interacting with the cellular receptor ACE2 are given at pH 4.6. The source of the three dimensional structures of these proteins are explained in the text and referred as $RBD1_{wt}$, $RBD2_{wt,}$, $RBD2_{wt'(6VSB),}$and $RBD2_{wt'(6VXX)}$, respectively. The plots for $RBD1_{wt}$ and $RBD2_{wt,}$ correspond to the same ones already given in Figure 2. Salt concentration was fixed at 150 mM. Simulations started with the two molecules placed at random orientation and separation distance. Results for SARS-CoV-1, SARS-CoV-2 (wt), SARS-CoV-2 (wt with RBD from PDB id 6SVB), and SARS-CoV-2 (wt with RBD from PDB id 6SXX) are shown as black, red, dark blue, and marron continuous lines, respectively.

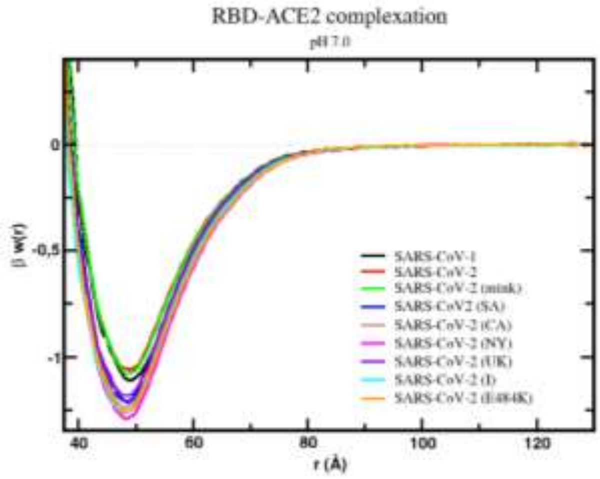

a.

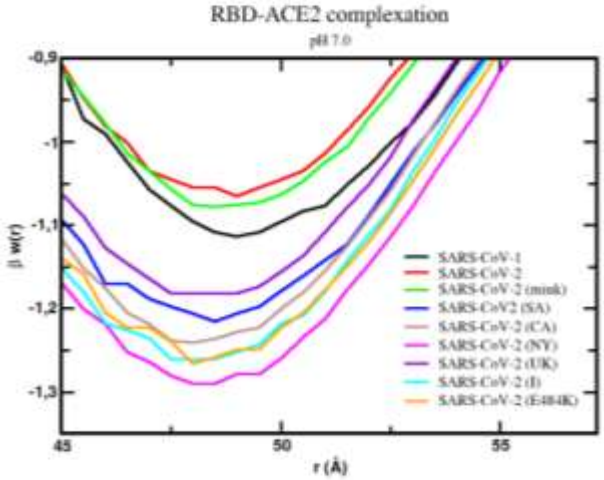

b.

Supplementary Figure 6. Free energy profiles for the interaction of RBD proteins with the cellular receptor ACE2. The simulated free energy of interactions [**β***w*(*r*)] between the centers of the RBD proteins from SARS-CoV-1, SARS-CoV-2 (wt), SARS-CoV-2 (mink), SARS-CoV-2 (SA), SARS-CoV-2 (CA), SARS-CoV-2 (NY), SARS-CoV-2 (UK), SARS-CoV-2 (I), and SARS-CoV-2 (E484K) and the cellular receptor ACE2 are given at pH 7.0. The source of the three-dimensional structures of these proteins are explained in the text and referred as $RBD1_{wt}$, $RBD2_{wt}$, $RBD2_{m}$, $RBD2_{SA}$, $RBD2_{CA}$, $RBD2_{NY}$, $RBD2_{UK}$, $RBD2_{I}$, and $RBD2_{E484K}$, respectively. Salt concentration was fixed at 150 mM. Data for the complexes with the wildtype proteins ($RBD1_{wt}$-ACE2 and $RBD2_{wt}$-ACE2) was given before (43). Simulations started with the two molecules placed at random orientation and separation distance. Results for SARS-CoV-1, SARS-CoV-2 (wt), SARS-CoV-2 (mink), SARS-CoV-2 (SA), SARS-CoV-2 (CA), SARS-CoV-2 (NY), SARS-CoV-2 (UK), SARS-CoV-2 (I), and SARS-CoV-2 (E484K) are shown as black, red, green, blue, dark purple, pink, light purple, cyan, and orange continuous lines, respectively. (a) *Left panel*: Full plot. (b) *Right panel*: The well depth region of the β*w*(*r*) for each studied complex.

1.1.3 Key-mutations

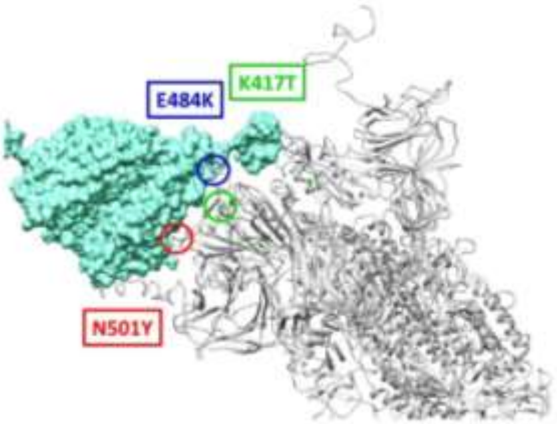

a.

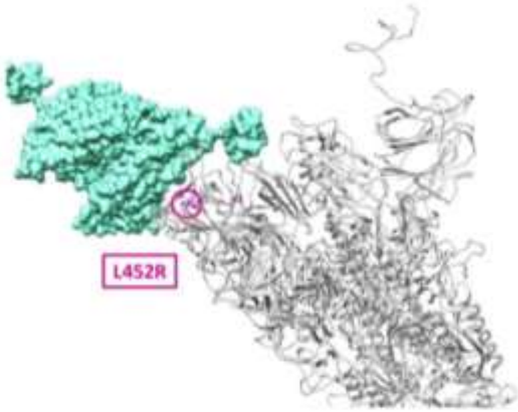

b.

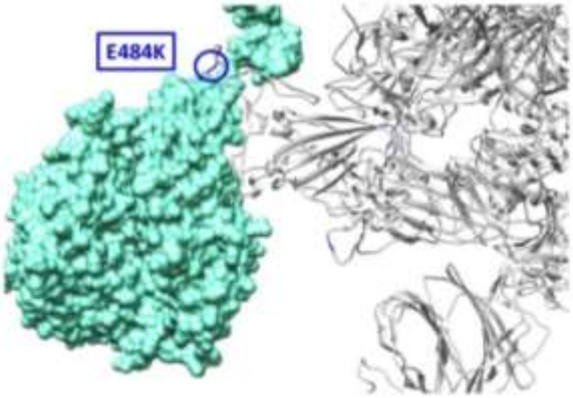

c.

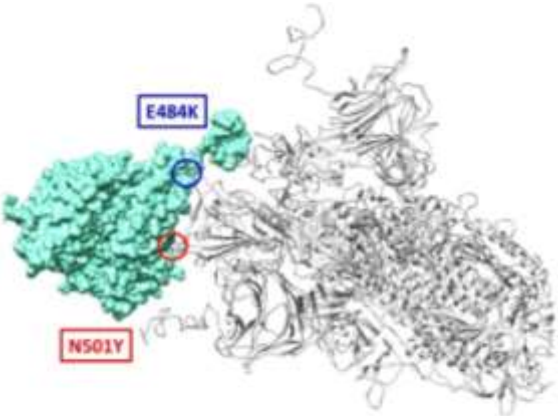

d.

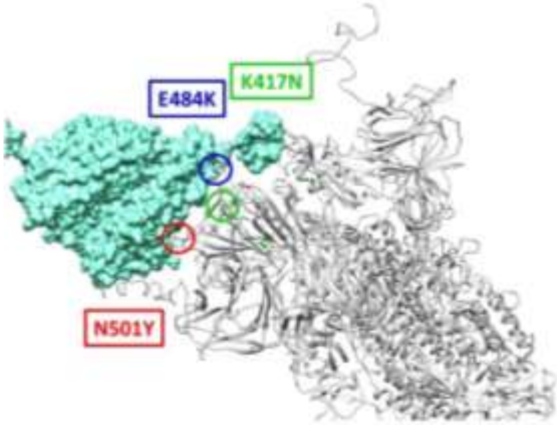

e.

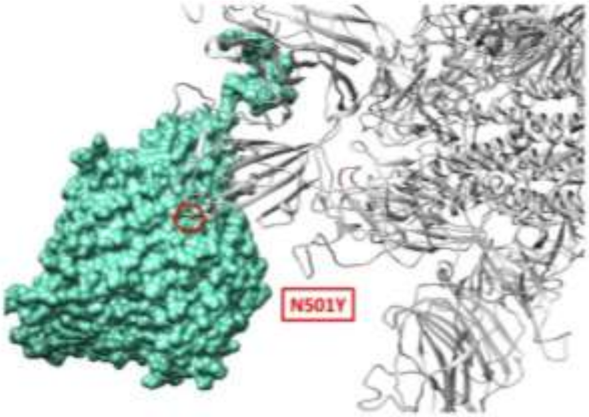

f.

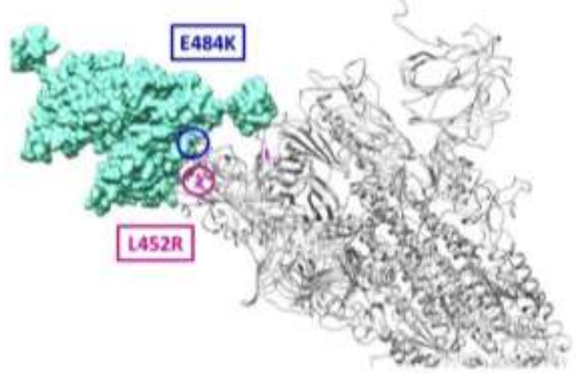

g.

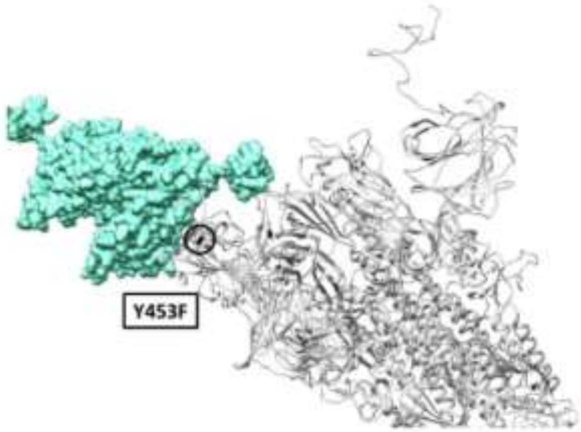

h.

Supplementary Figure 7. Molecular structures of the different variants of SARS-CoV-2 Spike complexed with ACE2. The aquamarine surface represents the receptor ACE2 and the grey ribbon represents the Spike homotrimer protein (PDB id 7A94). For better visualization of the epitope-paratope interface, the Spike was maximized and the ribbon was partially represented and the key-mutations for each variant were highlighted. The 8 images represent the variants: (a) Brazilian P.1, (b) Californian, (c) isolated E484K mutations, (d) the New York, (e) South-African, (f) UK variant, (g) Indian double mutations and (h) SARS-CoV-2 mink-associated variant strain. The Spike residue N501 is shown in red, E484 in blue, K417 in green, L452 in magenta and Y453 in black. The image was generated by UCSF Chimera (73). All used RBD structures are available at Zenodo (https://doi.org/10.5281/zenodo.4780600).

1.1.4 Main Conclusions

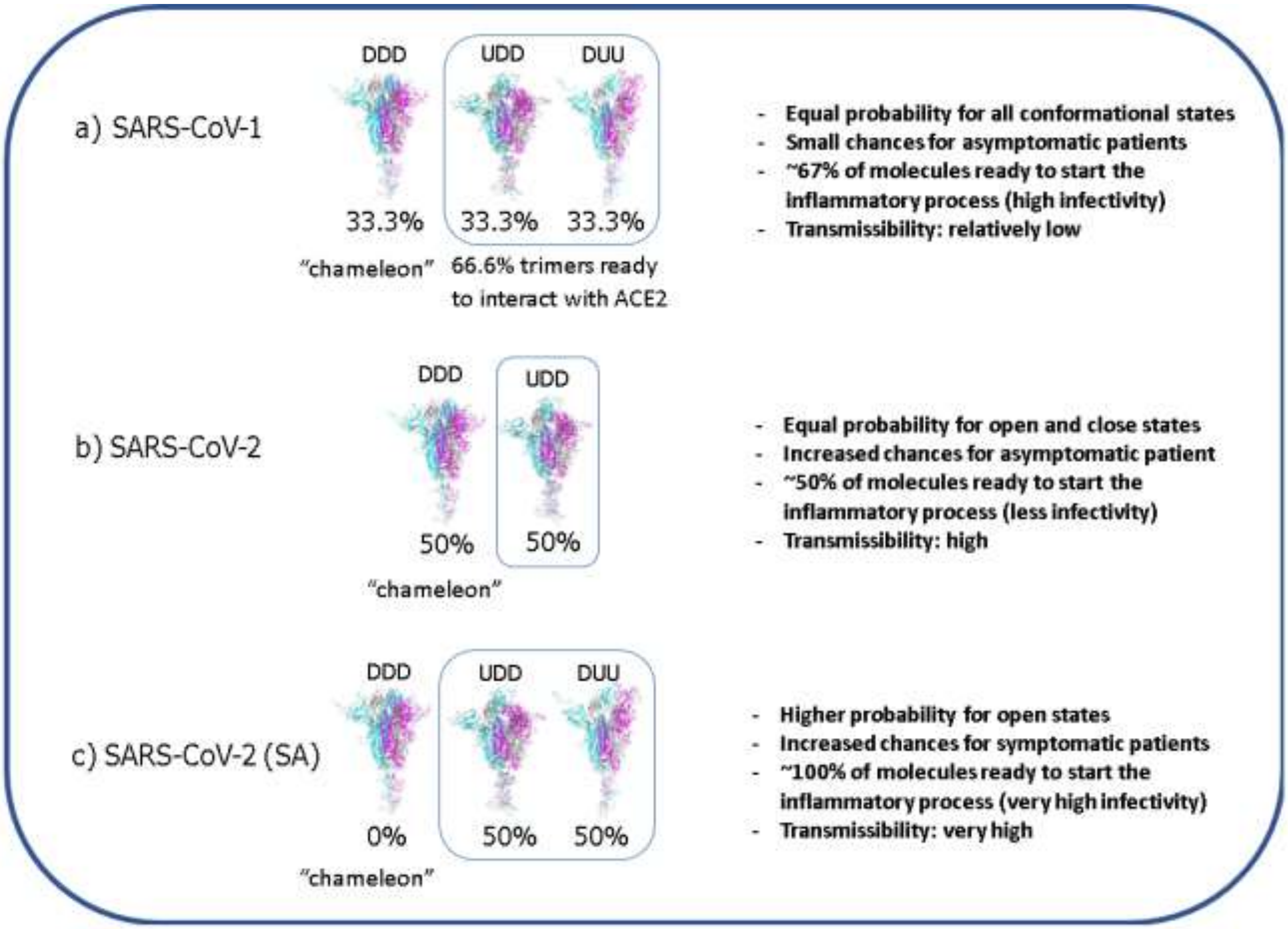

Supplementary Figure 8. Schematic representation for the main conclusions. The three sets show the probability that SARS-CoV-1 (a), SARS-CoV-2 (b) and SARS-CoV-2 (SA) (c) have for all conformational states (DDD, UDD and DUU) at the highest stability conditions and their possible relations with clinical aspects. The illustrative images of the spike homotrimers were generated by UCSF Chimera (PDB id 6VXX, 6VSB and 7A93 for the states DDD, UDD and DUU, respectively) (73).

1.2 Supplementary tables

Supplementary Table 1. Main physical chemical properties of the RBDs of different new variants. These results were obtained by the FPTS at physiological salt concentration. See the text for more details.

	Charges numbers for different protein structures							
	$RBD2_{wt}$	$RBD2_{m}$	$RBD2_{SA}$	$RBD2_{BR}$	$RBD2_{CA}$	$RBD2_{NY}$	$RBD2_{UK}$	$RBD2_{I}$
pH 4.6	5.5	5.5	6.2	6.2	6.4	7.1	5.5	7.0
pH 7.0	2.2	2.2	3.2	3.2	3.1	4.1	2.1	4.1
	Dipole moment numbers for the same structures							
pH 4.6	35	35	65	65	40	71	35	53
pH 7.0	31	31	82	82	43	90	31	71

Supplementary Table 2. Detailed data for the mapping of EE for the RBD at different conformational states of the spike homotrimer. The EE were predicted by the PROCEEDpKa method for the RBD of SARS-CoV-2 wt out of the homotrimer (as given in Figure 3). Exposed and hidden residues were identified by the on-line server "PDBePISa" (90) with default options. PDB ids 6VXX and 6VSB were used in this analysis.

A) EEs exposed to the solvent and available to directly interact with the receptor ACE2 for chain A of Spike RBD (PDB id 6VSB).

Spike RBD EE - Chain A (NEE=32) (NHE=6) (*up*)			
E340	K386	D427	E465
R346	D389	D428	R466
Y351	Y396	D442	D467
R355	R403	K444	Y473
K356	D405	Y449	Y489
R357	R408	Y451	Y495
Y365	K417	Y453	Y505
Y369	D420	R457	Y508
K378	Y421	K458	R509
Y380	Y423	K462	H519

B) EEs exposed to the solvent and available to directly interact with the receptor ACE2 for chain B of Spike RBD (PDB id 6VSB).

Spike RBD EE - Chain B (NEE=28) (NHE=11) (*down*)			
E340	K386	D427	E465
R346	D389	D428	R466
Y351	Y396	D442	D467
R355	R403	K444	Y473

K356	D405	Y449	Y489
R357	R408	Y451	Y495
Y365	K417	Y453	Y505
Y369	D420	R457	Y508
K378	Y421	K458	R509
Y380	Y423	K462	H519

C) EEs exposed to the solvent and available to directly interact with the receptor ACE2 for chain C of Spike RBD (PDB id 6VSB).

Spike RBD EE - Chain C (NEE=30) (NHE=10) (*down*)			
E340	K386	D427	E465
R346	D389	D428	R466
Y351	Y396	D442	D467
R355	R403	K444	Y473
K356	D405	Y449	Y489
R357	R408	Y451	Y495
Y365	K417	Y453	Y505
Y369	D420	R457	Y508
K378	Y421	K458	R509
Y380	Y423	K462	H519

D) EEs exposed to the solvent and available to directly interact with the receptor ACE2 for chain A of Spike RBD (PDB id 6VXX).

Spike RBD EE - Chain A (NEE=20) (NHE=20) (*down*)			
E359	K405	D446	E484

R365	D408	D447	R485
Y370	Y415	D461	D486
R374	R422	K463	Y492
K375	D424	Y468	Y508
R376	R427	Y470	Y514
Y384	K436	Y472	Y524
Y388	D439	R476	Y527
K397	Y440	K477	R528
Y399	Y442	K481	H538

E) EEs exposed to the solvent and available to directly interact with the receptor ACE2 for chain B of Spike RBD (PDB id 6VXX).

Spike RBD EE - Chain B (NEE=24) (NHE=15) (*down*)			
E359	K405	D446	E484
R365	D408	D447	R485
Y370	Y415	D461	D486
R374	R422	K463	Y492
K375	D424	Y468	Y508
R376	R427	Y470	Y514
Y384	K436	Y472	Y524
Y388	D439	R476	Y527
K397	Y440	K477	R528
Y399	Y442	K481	H538

F) EEs exposed to the solvent and available to directly interact with the receptor ACE2 for chain C of Spike RBD (PDB id 6VXX).

Spike RBD EE - Chain C (NEE=25) (NHE=15) (*down*)			
E359	K405	D446	E484
R365	D408	D447	R485
Y370	Y415	D461	D486
R374	R422	K463	Y492
K375	D424	Y468	Y508
R376	R427	Y470	Y514
Y384	K436	Y472	Y524
Y388	D439	R476	Y527
K397	Y440	K477	R528
Y399	Y442	K481	H538

Legend

(light blue)	Solvent-accessible residues
(blue)	Inaccessible residues
(orange)	Interfacing residues (AxB)
(yellow)	Interfacing residues (AxC)
(green)	Interfacing residues (BxC)
**NEE**	Number of exposed epitopes
**NHE**	Number of hidden epitopes (due to the interface with another chain)
